# 我如何诊断和治疗自身免疫性溶血性贫血

**DOI:** 10.3760/cma.j.issn.0253-2727.2022.11.005

**Published:** 2022-11

**Authors:** 蓉 付, 虹 于

**Affiliations:** 天津医科大学总医院，天津 300052 Department of Hematology, Tianjin Medical University General Hospital, Tianjin 300052, China

自身免疫性溶血性贫血（AIHA）是由于机体免疫功能异常，导致B细胞功能亢进产生针对自身红细胞的抗体，红细胞吸附自身抗体和（或）补体，致使红细胞破坏加速、寿命缩短的一组溶血性贫血。AIHA的年发病率为（1.0～3.0）/10万，患病率为17/10万[1]。女性稍多，中位发病年龄50岁。法国一项研究显示18岁以下儿童的年发病率为0.81（95％ *CI* 0.76～0.92）/10万[2]。

AIHA是一组异质性疾病，需要进行全面的实验室检查，以明确诊断并确定亚型。AIHA依据病因明确与否，分为继发性和原发性；根据自身抗体与红细胞最适反应温度，分为温抗体型（wAIHA）、冷抗体型（cAIHA）和混合型；依据红细胞自身抗体检测结果，分为自身抗体阳性型和自身抗体阴性型。继发性wAIHA占50％。wAIHA占所有病例的60％～70％，冷凝集素病（CAD）占20％～25％，混合型占5％～10％，非典型AIHA占10％，阵发性冷血红蛋白尿（PCH）占1％～3％。80％～90％的成人AIHA和75％的儿童AIHA是wAIHA。wAIHA中的自身抗体是泛凝集素，针对几乎所有个体的红细胞上Rh复合体上的蛋白质和红细胞膜上的糖蛋白抗原。wAIHA的病理机制呈现多样性，包括细胞外溶血、脾巨噬细胞清除IgG包裹的红细胞、抗体依赖性细胞介导的细胞毒性和补体激活。

不同亚型AIHA具有不同的临床特征及治疗反应。病程多呈慢性且反复发作，仅30％的患者通过初始治疗实现持久缓解。笔者结合1例典型病例，对AIHA的诊断和治疗全程管理进行探讨，供借鉴。

一、典型病例

男，44岁。因“头晕乏力1个月”就诊。否认家族史，体检示贫血貌、轻度黄疸和脾肿大。入院血常规：WBC 4.08×10^9^/L，淋巴细胞比例33.3％，PLT 248×10^9^/L，HGB 72 g/L，网织红细胞比例（Ret）7.8％；血清LDH 359 U/L，间接胆红素（IBIL）19.2 µmol/L；血浆结合珠蛋白（HP）0.185 g/L，游离血红蛋白（F-HB）47.4 mg/L，直接抗人球蛋白试验（DAT）IgG1阳性，Coombs试验IgG阳性（效价1∶128，积分52），Coombs试验C3阳性（效价1∶256，积分72）；铁蛋白>2000 µg/L，叶酸>20 µg/L，维生素B_12_ 467.3 ng/L，促红细胞生成素（EPO）>750 U/L；肾功正常，血、尿游离轻链正常，血清蛋白电泳、免疫固定电泳阴性；EB病毒、巨细胞病毒、微小病毒B19、乙型肝炎病毒（HBV）、丙型肝炎病毒（HCV）均阴性；风湿免疫全项均阴性；胸腹CT示脾大，余未见异常；骨髓涂片示红系增高、粒系和巨核系增生，骨髓活检示增生活跃、未见淋巴细胞和浆细胞增多；IgH、TCR重排均阴性，流式细胞术淋巴免疫分型、骨髓增生异常综合征（MDS）表型未见异常，未发现淋巴组织增生性疾病（LPD）证据。诊断为原发性wAIHA。

口服泼尼松1 mg·kg^−1^·d^−1^后溶血逐渐减轻，在第4周达到完全缓解（CR）后泼尼松逐渐减量，于CR后3个月停药。停药后5个月出现第一次复发，口服泼尼松仍有反应，但呈现依赖性。故给予每周利妥昔单抗100 mg连续4周治疗并将泼尼松逐渐减量至10 mg/d，患者HGB 113 g/L，Ret 4％，LDH 210 U/L，IBIL 8.6 µmol/L，达到部分缓解（PR）。利妥昔单抗治疗后23个月因感染出现第二次复发，HGB 73 g/L，Ret 5.7％，LDH 286 U/L，IBIL 35.3 µmol/L，DAT阳性，叶酸8.96 µg/L，维生素B_12_ 457.4 ng/L，红细胞寿命26 d；IgG 5.79 g/L，C3 375 mg/L，C4 141 mg/L；CD4/CD8 0.65；骨髓象：增生活跃，红系前体细胞增多，粒巨增生，MF-0级；淋巴免疫分型、MDS表型未见异常；PET-CT：体部未见肿瘤征象，扫描范围内骨髓代谢弥漫增高。全外显子组测序（WES）未发现已知的致病性突变及潜在的遗传缺陷。诊断为复发/难治（R/R）原发性wAIHA。予输血2 U，静脉甲泼尼龙联合丙种球蛋白（IVIg）治疗5 d，辅以碱化水化、护肝利胆、抗感染、降糖、抗凝等，后予环孢素A（CsA）75 mg每12 h 1次治疗，患者再次达到PR。目前仍在规律治疗随访中。

二、AIHA的诊断流程

首先，判断是否存在溶血，所有患者均应检测HP、LDH、Ret、TBIL、红细胞寿命及外周血涂片。HP和LDH常能提示溶血的存在，LDH升高和HP<250 mg/L对溶血的特异性达95％。Ret通常增高，但37％的患者在诊断时网织红细胞计数减低[3]。IBIL在血管外溶血时升高明显、LDH在血管内溶血时升高明显，但均非特异。红细胞寿命检测证实红细胞存活时间缩短。血涂片可见多色性、微球形细胞、Howell-Jolly小体、有核红细胞，有助于与血栓性微血管病变相鉴别。

其次，判断是否存在因过度免疫反应产生的针对自身红细胞抗体，DAT是诊断AIHA的基石。DAT是应用单特异性抗血清检测患者红细胞上是否存在抗体和（或）补体，其特异性高但灵敏度低。5％～10％的AIHA患者可能是DAT阴性，原因是存在受洗涤液影响的低亲和力IgG自身抗体，或存在低于测试阈值的红细胞结合抗体，或非IgG抗体而是IgA或IgM自身抗体。为提高检测阳性率可采用流式细胞术、酶联放射性标记试验、丝裂原刺激的增强DAT，这些方法灵敏度高但特异性低，容易出现假阳性，假阳性率为31％～55％[2]。DAT阴性，IgA和IgM-wAIHA通常被定义为非典型AIHA[2]。

最后，应全面筛查其他潜在疾病。成人wAIHA患者中约50％是由潜在疾病引起或与之相关，称为继发性wAIHA。引起继发性wAIHA的疾病常见的有淋巴组织增生性疾病（LPD）、自身免疫性疾病、免疫缺陷、移植、实体瘤，也可与某些药物相关[4]。有研究显示99例原发性AIHA应用嗜水气单胞菌溶素变异体（Flaer）检测WBC和RBC，37例（37.4％）患者粒细胞上存在微小PNH克隆（<10％），中位克隆比例为0.2％（0.03％～85％）。PNH阳性的AIHA患者LDH水平更高，溶血更显著，血栓形成风险增加，IFN-γ和IL-17水平降低[5]。某些微生物如新型冠状病毒COVID-19、严重急性呼吸综合征冠状病毒2（SARS-CoV-2）、布鲁士菌、巴贝虫病等也可引起AIHA[6]。药物也是导致AIHA的重要因素，超过150种药物可以诱发AIHA，药物诱发的AIHA占全部AIHA的10％。所以所有AIHA患者均应行风湿免疫、病原微生物检测；应详细询问个人史及用药史（含疫苗接种）；行影像学、骨髓细胞学、骨髓活检、流式细胞术等检测。若冷凝集素阳性，疑为CAD患者，应常规筛查血清蛋白电泳、免疫固定电泳等。原发性免疫缺陷相关基因（TNFRSF6、CTLA4、STAT3、PIK3CD、CBL、ADAR1、LRBA、RAG1和KRAS）突变可出现于40％的Evans综合征（ES）[7]，Sanger分子测序可早期识别及诊断。

三、AIHA治疗

继发性AIHA，需要积极治疗原发病，并考虑原发疾病所处的阶段和活动性，采取不同的治疗方案。原发性AIHA，对于轻度和部分代偿性溶血性贫血（HGB>100 g/L）患者，权衡利弊后，可“观察和等待”。启动治疗的指征是症状性贫血。治疗目标是提升患者血红蛋白水平，改善症状，提高生活质量。

（一）急危重症处理

1. 输血：应遵循“不禁忌，但非必要则避免”的原则[2]。对于急性、重症患者，进行扩展表型的交叉配型排除同种异体抗体，可予红细胞缓慢输注，同时注意碱化、水化利尿。

2. 急危重症治疗：指HGB<60 g/L和（或）血流动力学不稳定患者，其死亡率高达30％～57％。可予静脉甲泼尼龙100～200 mg/d，连用7～10 d或250～1 000 mg/d，连用1～3 d；输血1 U/d；存在感染的情况下可予IVIg 0.4 g·kg^−1^·d^−1^，连用5 d；一周内无效可早期使用利妥昔单抗375 mg/m^2^，每周1次，治疗4周；也可予血浆置换（PEX）；若有血栓高危因素，且无禁忌证，可给予低分子肝素预防血栓。

（二）一线治疗

糖皮质激素为wAIHA的一线治疗药物。成年患者口服1.0～1.5 mg·kg^−1^·d^−1^泼尼松的缓解率为75％～80％，但仅有20％～30％的患者在糖皮质激素减量/停药后持续缓解[1]。替代性糖皮质激素如静脉甲泼尼龙、地塞米松亦具有治疗作用，但在wAIHA缺乏头对头临床研究。《AIHA的诊断和治疗中国专家共识》中推荐泼尼松口服剂量为0.5～1.5 mg·kg^−1^·d^−1^，最佳剂量和减量时间表仍证据有限。

既往研究显示，53例初诊wAIHA患者接受1～2 mg·kg^−1^·d^−1^泼尼松治疗，治疗时间为（15±3）个月，46例有治疗反应，反应时间为（25±15）d，2 mg·kg^−1^·d^−1^泼尼松治疗患者中位反应时间为7（2～21）d；患者中20％继发糖尿病，10％原有糖尿病恶化，10％发生骨质疏松伴骨折，4％发生股骨头坏死[8]。

糖皮质激素用至红细胞比容大于30％或HGB大于100 g/L以上可考虑减量[2]，或因累积的红细胞比容不良反应不能耐受，必须减量。一项研究显示，若红细胞比容在2个月内逐渐减量至10 mg，在6个月内停药，易导致疾病复发[9]。故建议在几周内将泼尼松减至20～30 mg，后每月递减2.5～5 mg。减量过程中应同时监测HGB和溶血指标，如HGB低于100 g/L且有溶血指标阳性，则延迟减量，持续CR至少3个月，考虑停用糖皮质激素。

关于利妥昔单抗用于AIHA的一线治疗有两项前瞻性随机对照试验。一项研究中，患者随机分为单药泼尼松组、泼尼松联合利妥昔单抗组，每组32例，利妥昔单抗每周375 mg/m^2^，连续4周，泼尼松联合利妥昔单抗组12个月有效率显著高于单药泼尼松组（75％对36％，*P*＝0.003），随访36个月，泼尼松联合利妥昔单抗组无复发生存率较高（70％对45％）[10]。另一项随机双盲对照研究中，接受标准剂量泼尼松治疗的患者随机接受安慰剂或利妥昔单抗1 g（固定剂量）第1、15天，泼尼松联合利妥昔单抗组在12个月（75％对31％，*P*＝0.032）和24个月（63％对19％，*P*＝0.011）的有效率均显著高于对照组[11]。两项研究均未发现利妥昔单抗组出现严重不良反应。

（三）二线治疗

泼尼松1 mg·kg^−1^·d^−1^治疗3周无应答的糖皮质激素耐药者、使用推荐剂量治疗4周未达到PR或糖皮质激素停药后复发者，可接受二线治疗。

利妥昔单抗是wAIHA国际专家共识建议的唯一二线治疗方法。在包括409例患者的21项研究的Meta分析中显示，利妥昔单抗治疗复发或难治wAIHA的总有效率为79％，CR率42％，中位反应时间为3～6周，3年持续有效率为60％[12]。标准方案为利妥昔单抗每周375 mg/m^2^连续4周，或1 g（固定剂量）第1、15天的方案，两种给药方案总剂量相似，应答率相似[12]。对于非重型和老年患者可予小剂量方案，每周100 mg（固定剂量）连续4周，在减少不良反应的同时并不降低疗效，24个月有效率为100％[13]。天津医科大学总医院单中心回顾性研究显示，22例难治性AIHA使用小剂量利妥昔单抗（每周100 mg，连续4周）方案，有效率为95.2％，CR率为50.0％，达效迅速，各项溶血指标均获得恢复，随访24个月有效率为100.0％，CR率为40％，患者均耐受良好[14]。但多项研究显示，对治疗有反应的患者中超过30％可能在3年后复发[12]，复发患者距上次给药时间超过12个月，可再次使用利妥昔单抗。

利妥昔单抗使用前应常规检测HBV、HCV、HIV和结核杆菌。对于抗HBC和（或）抗HBS抗体阳性且未接种乙肝疫苗患者，建议使用恩替卡韦或拉米夫定预防18个月[2]。

（四）三线治疗

1. 脾切除术：原发性wAIHA患者，脾切除术有效率为70％，CR率为40％[1]。一项对15例wAIHA患者脾切除的研究表明，随访4.5年，缓解率为90％，复发率为30％[15]。另一项对37例脾切除术患者的研究显示，中位随访33.1个月，缓解率为81％[16]。三分之一的wAIHA患者在脾切除术后复发，长期缓解率（≥10年）尚不清楚。伴抗磷脂综合征的患者应避免脾切除术。尚无指标能预测脾切除的疗效，但有效性与疾病持续时间、对糖皮质激素的反应或脾肿大的程度无关。患者在术后第1年易发生严重感染，对于年龄大于65岁，有心肺病史、血栓栓塞病史、丙型肝炎、潜在的免疫缺陷、淋巴组织增生及系统性自身免疫性疾病的患者，不推荐行脾切除术。目前脾切除术使用率已下降至10％[2]。

2. 硫唑嘌呤：2～2.5 mg·kg^−1^·d^−1^的有效率为71％（22/31，所有类型AIHA），60％（9/15，wAIHA），56％（5/9，wAIHA）[1]–[2]。

3. CsA：2.5 mg/kg，每日两次，有效率为58％（7/12，所有类型AIHA）[1]。一项研究显示10例R/R wAIHA患者接受CsA 3～5 mg·kg^−1^·d^−1^治疗，3个月有效率为40％，12个月有效率为83％[17]。

4. 霉酚酸酯：500～1 000 mg，每日两次，小系列病例报告的有效率分别为100％（4/4，所有类型AIHA）、25％（1/4，wAIHA）和67％（AIHA和CAD）[1]–[2]。

5. 西罗莫司：mTOR抑制剂，已成功用于治疗移植后AIHA、自身免疫性淋巴细胞增生综合征（ALPS）、儿童难治性原发wAIHA和ES[18]–[19]。国内研究显示14例原发性R/R AIHA及12例原发性R/R ES患者接受西罗莫司1～3 mg/d治疗，AIHA的CR率与有效率分别为57.1％、85.7％，ES分别为25.0％、83.3％，中位应答时间为2（2～5）个月，CR患者均在治疗6个月内达到最佳疗效[20]。天津医科大学总医院前期数据显示17例难治性AIHA患者接受西罗莫司治疗，有效率为94.1％，CR率为52.9％；中位起效时间34.5（2～363）d，中位疗效维持时间309（22～718）d，16例患者在随访期间无复发。

6. IVIg：IVIg可加速内源性IgG（致病性自身抗体）的清除，对部分AIHA患者有效。一项研究显示予0.4～0.5 g·kg^−1^·d^−1^ IVIg治疗5 d，32％的患者有反应，应答可持续3周以上[2]。国内前瞻性观察IVIg治疗组有效率为90％，12个月复发率为15％，较传统方案更能迅速控制病情，同时在短期内增强和巩固糖皮质激素的疗效[21]。

（五）后线治疗

R/R AIHA患者目前尚无标准治疗方案，国内外专家共识均推荐首选临床试验[2],[4]。Barcellini等[22]汇总了目前正在进行的关于wAIHA的临床试验或已在病例报道中描述的靶向治疗研究。其他可选择的药物如下：

1. 环磷酰胺（CTX）：一项研究报道了13例wAIHA患者每月静脉注射CTX 1 g，治疗4个月有效率为82％，治疗6个月有效率为100％、CR率为47％[1]。天津医科大学总医院回顾性研究29例难治性AIHA使用静脉CTX 1 g（固定剂量），每10 d 1次，连续3次，12个月有效率为90％，24个月有效率为100％，CR率为40％，不良反应少、耐受良好[14]。最新一项回顾性研究显示16例R/R wAIHA患者接受利妥昔单抗、CTX和地塞米松联合治疗，中位2个周期有效率达94％，中位反应持续时间为9.8个月，原发、继发及接受过利妥昔单抗亚组疗效相似[23]。

2. 达那唑：口服200 mg，每天3次，但在难治患者中疗效不显著。

3. 硼替佐米：1.3 mg/m^2^，每周1次，1～4周，或每周两次（d 1、4、8、11），1～3个周期。有报道显示4例难治wAIHA患者中3例有效，8例多重难治wAIHA患者中6例有效[24]。wAIHA中利妥昔单抗联合硼替佐米的有效率为70％[25]。

4. CD38单抗：有报道达雷妥尤单抗治疗3例难治性移植后AIHA患者，2例反应良好[22]。在非造血干细胞移植成年wAIHA患者应用达雷妥尤单抗16 mg/kg每周1次治疗，使糖皮质激素依赖和利妥昔单抗难治患者获得5个月无糖皮质激素治疗（NCT05004259）。

5. 布鲁顿酪氨酸激酶（BTK）抑制剂：施均教授团队曾报道2例R/R原发性AIHA在接受伊布替尼治疗后获得CR伴血细胞计数未完全恢复（CRi）[26]。近期全球多中心对接受BTK抑制剂治疗的cAIHA患者进行回顾性研究，4例原发性CAD、11例继发性冷凝集素综合征（CAS）（5例继发于华氏巨球蛋白血症、5例继发于慢性淋巴细胞白血病、1例继发于小淋巴细胞淋巴瘤），中位接受3（0～5）线治疗，共7例患者检测MYD88突变，其中3例华氏巨球蛋白血症患者MYD88 L265P突变阳性。12例达CR，1例PR，12例溶血指标改善，且不良反应可控，显示在MYD88野生型的CAD中有效[27]。目前有多项临床试验正在招募包括Ibrutinib（NCT03827603、NCT04398459）、Acalabrutinib（NCT04657094）及Rilzabrutinib（NCT05002777）。天津医科大学总医院正在开展100 mg/d奥布替尼单药治疗R/R AIHA、Evans综合征的单中心前瞻性临床研究，前期小样本研究显示BTK抑制剂治疗可能成为一种选择。

6. 补体靶向药物：①Eculizumab，抗C5单克隆抗体，13例CAD患者治疗后中位LDH从572 U/L下降至334 U/L、中位HGB从93.5 g/L增加至101.5 g/L、输血需求显著降低、生活质量改善[28]。②Pegcetacoplan（APL-2），抑制C3活化（NCT03226678、NCT05096403）；③Sutimlimab（BIVV009、TNT009），特异性抑制C1s，Ⅰb期临床试验结果显示，10例CAD患者中有7例HGB增加20 g/L以上，第1周HGB中位升高16 g/L，6周内HGB中位升高39 g/L（NCT03347422、NCT05132127）；④ANX005，C1q抑制性分子（NCT04691570）；⑤Iptacopan（LNP023），靶向补体旁路途径（NCT05086744）；⑥Fostamatinib，靶向syk，17例AIHA患者中有效率为53％，中位HGB增加30 g/L，溶血指标改善，且反应得到维持，不良反应小（NCT04138927、NCT03764618）；⑦Nipocalimab，靶向FcRn（NCT04119050、NCT05221619），由天津医科大学总医院血液科牵头，全国多中心参与的一项随机、双盲、安慰剂对照（包括开放性扩展期）研究（MOM-M282-006）旨在评价Nipocalimab（M281）在wAIHA成人患者中的疗效和安全性正在开展；⑧Parsaclisinib，靶向PI3K（NCT03538041、NCT05073458）；⑨Alemtuzumab，靶向CD52，初步显示了在AIHA的疗效。

（六）其他治疗

1. EPO：低Ret与不良预后相关。近期一项国际多中心研究显示，51例AIHA患者，40％的患者存在红系生成障碍，AIHA患者中位EPO 32（9.3～1 328）U/L，用rEPO单独或与其他药物联合治疗，1个月有效率为71％、12个月有效率为78％，且CR率随时间延长逐渐增加，治疗1个月中位HGB增加24（2～83）g/L，2例患者发生血栓栓塞事件（TE）。在AIHA诊断后第一年内开始rEPO治疗的患者有效率更高（85％对64％，*P*＝0.06），在原发性AIHA患者中观察到更好的反应（77％对44％）[29]。

2. PEX：去除致病性免疫复合物、循环自身抗体和活化补体。IVIg和PEX应被视为桥接疗法用于急性和重症患者。

四、常见不良反应和对症治疗

1. 感染：多中心回顾性研究显示，3级以上感染发生率为10％，主要是肺炎、呼吸功能衰竭、感染性休克[30]。患者接受大于20 mg/d糖皮质激素超过1个月，应给予甲氧苄氨嘧啶-磺胺甲恶唑预防肺孢子虫肺炎。

2. TE：两项大型研究显示，11％的AIHA患者发生血栓事件[30]。CAD、脾切除术后、抗心磷脂抗体阳性、狼疮抗凝物阳性患者均建议血栓预防。若有血栓高危因素，且无禁忌证，可给予低分子肝素预防血栓。

3. 糖皮质激素相关性骨质疏松症：泼尼松剂量大于7.5 mg/d超过3个月可致骨质疏松致骨折风险增加[30]。推荐摄入维生素D 800 U/d和钙700～1 200 mg/d，50岁以上的男性和绝经后女性加用双膦酸盐预防骨折，并应定期测量骨密度[1]–[2]。

4. 消化道出血：接受糖皮质激素治疗的患者上消化道并发症增加2.2～4.2倍，年龄大于60岁、既往胃及十二指肠溃疡病史、同时使用阿司匹林或非甾体抗炎药和血小板减少患者，推荐抗酸治疗[1]–[2]。

5. 造血原料缺乏：当溶血增加红细胞周转时，需求增加。血管外溶血可出现高铁蛋白血症, 但血管内溶血可导致铁丢失。据报道，不同病因的慢性AIHA患者均存在叶酸缺乏，推荐叶酸1～5 mg/d [1]–[2]并定期检测造血原料水平。

五、疗效评价

CR定义为HGB和溶血标志物（IBIL、LDH、HP和Ret）的正常化；PR定义为HGB≥100 g/L或至少增加20 g/L，有或没有溶血检测指标改善；无应答或复发定义为HGB<100 g/L或至少减少20 g/L伴溶血标志物的改变而降低。难治性AIHA定义为患者对至少3线治疗［包括脾切除术和（或）至少一种免疫抑制剂］无反应[2],[4]。

六、远期随访和管理

GIMEMA研究发现，AIHA相关死亡率为3％～4％，血栓形成、感染、多线治疗（4线以上）、脾切除术、血小板减少、急性肾功能衰竭使患者死亡风险增加[30]。此外，缓解的患者在随访过程中仍需高度警惕继发LPD或其他系统疾病可能，建议应定期行血清学、影像学等检查。

七、总结

综上所述，wAIHA是一种慢性、异质性、易复发且临床过程不可预测的疾病。无论是初诊还是R/R患者，均应全面评估溶血参数、骨髓特征、疾病性质等。应正确使用糖皮质激素并逐渐减量停药，可早期使用利妥昔单抗，适时考虑脾切除术，并全面评估R/R患者可能发展的疾病，实现从经验性到靶向化治疗策略的转变，以期减少复发，达到治愈。
